# Fur, Fin, and Feather: Management of Animal Interactions in Australian Residential Aged Care Facilities

**DOI:** 10.3390/ani12243591

**Published:** 2022-12-19

**Authors:** Wendy Newton, Tania Signal, Jenni A. Judd

**Affiliations:** 1 School of Health, Medical & Applied Sciences, Central Queensland University, 6 University Drive, Bundaberg, QLD 4670, Australia; 2 School of Health, Medical & Applied Sciences, Central Queensland University, Building 6, Bruce Highway, Rockhampton, QLD 4702, Australia; 3 Research Division, Central Queensland University, 6 University Drive, Bundaberg, QLD 4670, Australia

**Keywords:** animal-assisted intervention, animal-assisted activities, visitation animals, pet therapy, policy, long-term care facility, nursing home

## Abstract

**Simple Summary:**

Animals are seen frequently in Australian Residential Aged Care Facilities (RACF). Research has demonstrated that there are risks to residents and the animals involved, with few policies or guidelines to assist managers in developing RACF animal policies. Via survey and interview, we asked RACF managers how they managed the interactions between residents and animals in their facilities. While the majority of RACFs reported some level of policy, coverage was limited, potentially risking animal and/or resident safety. For example, many facilities had no policy around handwashing when handling animals, and many facilities allowed visitors to bring family pets onsite but had no rules to guide these visits. The results of this study suggest that RACF managers need access to guidelines for the inclusion of animals in their facilities to keep residents and animals safe.

**Abstract:**

Animal-assisted interventions (AAI) have been occurring in Australian Residential Aged Care Facilities (RACF) for more than 40 years and may relieve loneliness and improve quality of life. The presence of animals in RACF poses an inherent risk to residents and the animals involved. Little is known about the policies and guidelines for including animals in the Australian RACF. We anticipated that most RACFs would have some policies, but they may lack the detail necessary to keep humans and animals safe. Using an adapted survey, we surveyed and interviewed a small but representative sample of Australian RACF managers. The results demonstrated that RACF did have animal policies; however, the content regarding the need for hand washing, infection prevention, and animal welfare was lacking. Including unregulated family pets in RACF was an unexpected additional risk factor identified during data analysis. There is a need for national guidelines tied to the national aged care policy, which includes training and educational resources for RACF and AAI providers.

## 1. Introduction

Non-human animal (referred to hereafter as animal or pet) visits and animal-assisted interventions (AAI) have been occurring in Australian residential aged care facilities (RACF) and geriatric wards for some time. One of the first publications examining their effectiveness in the Australian context was Salmon, et al. [1]. AAI may relieve loneliness and isolation for RACF residents [2] and, when incorporated with a therapeutic intervention; e.g., occupational therapy and physiotherapy [3,4], improves quality-of-life outcomes. AAI, as defined by the International Association of Human–Animal Interaction Organizations (IAHAIO), is “a goal orientated and structured intervention that intentionally includes or incorporates animals…” [5] (p. 5). In contrast, animal-assisted activities (AAA) are a less formal AAI and include “…visiting companion animals for ‘meet and greet’ activities with residents in nursing homes” [5] (p. 5). AAA in various forms occur in Australian RACF, including visits by dog handler teams that have undergone specific training to enter RACF, visits by petting zoos, farmed fish, or live-in animals freely wandering the facility.

Australia is a country of pet owners, with 90% of Australians owning a pet at some time in their lives [6]. For elderly pet owners or animal lovers, moving to an animal-free RACF environment may increase trauma and exacerbate feelings of loss and isolation inherent to moving into aged care [7,8]. Equally, an older person relocating from their residence to an RACF may result in the re-homing, surrender, or, in the worst case, euthanasia of their much-loved companion animal if no suitable alternative is identified [9]. The International Association of Human–Animal Interaction Organizations (IAHAIO) Chicago Declaration outlines four resolutions. One resolution is to ensure the provision of appropriate animal interactions to residents of aged care facilities who desire such interaction [10], and many Australian RACFs do provide such access. From a regulatory perspective, Federal Australian Aged Care legislations allow residents to participate in social activities, obtain social support, and have the right to choose preferred activities [11]. Therefore, providing access to AAA (as a minimum) within RACF should be considered a “how do we include animals?” question rather than a “should we include animals?” question.

Bringing animals into RACF, however, has inherent risks for humans and animals, from obvious risks of trips, falls, bites, and scratches to welfare implications for visiting animals [12,13]. As RACFs continue to manage the fallout of the pandemic, focus on and concern about zoonoses (diseases transferred from animals to humans or vice versa) is heightened [14]. The presence of animals in RACF increases the risk for zoonotic transfer [15,16], and the characteristics of RACF, such as staffing, skill mix, and large numbers of frail older persons living in close contact, further elevate the risk [17]. Australian RACF provides federally subsidized full-time permanent or temporary care for older adults (aged over 65 years). The care provided includes, as a minimum, supplying a clean room, washing laundry, and supplying meals in a central dining area for more independent residents. For residents with complex health care needs, such as cancer, heart disease, and various forms of cognitive decline, facilities provide additional skilled nursing care on an as-needed basis [18]. In June 2021, there were 2705 Australian RACF’s comprising 933 for-profit facilities and 1772 not-for-profits, with 830 individual providers. Nearly two-thirds of facilities, 62.7%, are in ‘metropolitan areas’, with very few, 1.6%, in either remote or very remote areas. The remainder is distributed between regional centers, 7.8%, large rural towns, 8.5%, medium rural towns, 7.2% and small rural towns, 12.3%. Facility size varies, with 11.4% having less than 30 beds, 26.1% with 31–60 beds, 33% with 61–100 beds, 27.7% with 101–180 beds, and 1.8% over 181 beds (https://www.gen-agedcaredata.gov.au/Topics/Providers,-services-and-places-in-aged-care accessed on 15 February 2020). Therefore, RACFs are referred to in health promotion as a ‘setting for health’ (other examples might be a town or a workplace), or a place where environmental, organizational, and personal factors impact health and well-being [19].

Zoonotic diseases such as *Salmonella* spp., *Escherichia coli* (*E. coli*), and *Campylobacter* can cause severe gastroenteritis resulting in hospitalization or death [20,21] with antibiotic-resistant strains of these zoonoses more likely in animals fed raw meat diets [21,22]. The reverse (human to animal) transfer is also possible, e.g., where an animal licks either the environment or a person with a gastrointestinal infection, and the animal becomes sick or passes the bacteria to another human [15]. This reciprocal link between human and animal health within a shared environment or ‘setting for health’ fits with the One Health framework [23]. Similarly, the link between human and animal welfare within the shared environment fits within the One Welfare framework [24]. Before including animals in RACFs, consideration of the benefits and risks to humans and animals in the particular setting should drive the types of interactions provided (i.e., visiting or resident animals, appropriate spaces and species for the specific facility etc.) [25]. Therefore, comprehensive policies and procedures concerning the inclusion of animals in RACFs would seem essential [9,26].

Despite this, relatively little is known about the policies and guidelines that currently influence the provision of AAA in the Australian RACF. Limited international evidence; e.g., [27,28,29], suggests that RACFs frequently lack institutional policies or adequate policy content, leaving residents, staff, and animals with little protection from possible health, safety, and welfare issues [9,30]. Stull, Hoffman, and Landers [27] found that despite most facilities having institutional policies, their content did not address the variety of species present, staff training, or specific management needs for the different animal-ownership models. A systematic literature review by Newton, Signal, and Judd [9] examining current policies and guidelines for AAA in RACFs identifies seven key themes (animal policies as a requirement, animal behavioural assessment, animal health screening and feeding for raw diets, allergy and phobia screening, restricted zones, species restriction, and animal welfare) for developing comprehensive animal policies. The current study extends the work of Stull, Hoffman, and Landers [27] to the Australian Aged Care sector. RACF managers were asked about current policies and practices concerning animals within their facilities via a survey, and were invited to participate in a follow-up interview. Based on the findings of Stull, Hoffman, and Landers [27] and the seven themes that emerged from the systematic literature review by Newton, Signal, and Judd [9], we predicted that (i) most RACFs will have some animal policies but that (ii) those policies will lack the necessary content and detail to keep humans and animals safe.

## 2. Materials and Methods

### 2.1. Participants and Recruitment

A private database of 1097 Residential Aged Care Facility (RACF) managers and nursing directors across Australia was used to email an anonymous Qualtrics^®^ [31] survey link. After the initial recruitment email, reminders were sent after one week, six weeks and eight weeks. State-managed multipurpose facilities (i.e., combined hospital and aged care) were excluded from the mailout because these facilities fall under different legislation and must comply with State Health Department regulations [32]. A total of 31 RACF managers completed a 15-min survey (41 questions) on the Qualtrics^®^ [31] platform. Full demographic details are available in the Results. The survey was adapted and developed from the instrument used by Stull, Hoffman, and Landers [27], who provided their survey and permission to use and adapt it for this purpose. Australian nursing professionals with RACF and management experience scrutinized the revised survey, and their comments and advice assisted in editing items to fit the Australian context. Questions had forced responses where possible, and skip logic was used to reduce user fatigue. Participants also had the opportunity to leave and return to the survey when convenient. An optional link was provided at the end of the survey to enlist willing participants in a follow-up interview. One manager volunteered and participated in an hour-long semi-structured interview.

The survey was administered via Qualtrics^®^ [31] and was accessible from 3 July 2021 until 26 August 2021; ethical approval was gained from CQ University Human Research Ethics Committee (Project Number: 0000022749). Participants were offered the opportunity to participate in a random draw for one of four $50 gift vouchers as an incentive to participate. Two weeks after the survey opened, Australia experienced an outbreak of the Delta variant of COVID-19 and RACFs across the country went into lockdown. This outbreak, and associated health measures, continued to affect RACF in several Australian states and territories until after the survey closed.

### 2.2. Measures

Survey data were exported into SPSS Statistics 26 [33] for analysis. Given the relatively small sample size, most analyses were descriptive with basic non-parametric tests (Chi^2^) to supplement where possible. The semi-structured interview was recorded using the ZOOM platform and transcribed using Otter.ai. The participant’s facility remoteness was measured using the Modified Monash Model used for the survey, and the interview was de-identified during transcription. Two authors independently deductively coded the interview transcript against the seven themes identified in [9] before cross-checking and agreeing on coding with the third author.

RACF data were categorized using three demographics chosen because of their stability over time [34]. These were bed numbers [0–30, 31–60, 61–100, 101–180 or >180], funding source [not for profit vs. for profit], and regionality [1 Metropolitan, 2 Regional centers, 3 Large rural towns, 4 Medium rural towns, 5 Small rural towns, 6 Remote, or 7 Very remote]. Respondents were asked about the presence of animals (visiting or owned by the RACF or resident/s) in their RACF. If no animals were present in their facility, they were asked whether there was any interest in providing animal interactions.

For facilities that included interactions with animals, data were collected on the types of interactions, the presence of policies, the species permitted to interact with the residents, and any restrictions placed on the visiting animal. A three-point Likert scale (yes, no, or unsure) was used to gather responses to scenario-based questions examining why visiting animals might be excluded, e.g., recent vomiting or diarrhea. Additional questions focused on the types of animals permitted as live-in animals (i.e., living full-time in the facility) and whether they were facility or resident-owned.

## 3. Results

Forty-six respondents started the survey, and after removing incomplete entries, 31 RACF managers informed this study. While the largest group of participants were managers of facilities located in metropolitan areas (39%), all bar ‘very remote’ locations were represented in the sample. For context, very remote facilities constitute less than 0.5% (*n* = 10) of all Australian RACFs [35]. Demographic details, including funding type, remoteness, and number of beds/facility size, are presented in Table 1. Facility size and remoteness were significantly related (Chi^2^=33.697, *p* = 0.028, Likelihood Ratio used due to the number cells with counts <5), with most metropolitan respondents from facilities in the 31–60 bed range. Smaller facilities (0–30 beds) were in the small rural town or remote areas. Funding source and remoteness were statistically independent (*p* > 0.05); however, we noted a trend with a higher percentage of non-profit facilities in more remote areas.

Managers of RACFs were asked a series of policy questions using three domains of animal inclusion: facility-owned, resident-owned, or visiting. A majority of facilities indicated having some form of policy regarding facility-owned (87%), resident-owned (81%), or visiting (84%) animals. However, as shown in Table 2, coverage was relatively low when managers responded to questions about specific guidelines within these policies, such as handwashing, management of bites or injuries, restricted areas, and allowed species. Please note, percentages were calculated based on a different number of respondents in the first two columns of Table 2, due to three facilities not allowing resident or facility-owned animals. This is outlined in the annotation below the table. Notably, less than half of the institutions had policies that included guidance on hygiene practices for staff and residents who interact with visiting animals. Less than 40% stipulated specific areas visiting animals could/could not go.

### 3.1. Visiting Animals

Of the 31 respondents, 26 facilities (84%) allowed animals to visit. Species visiting were dogs (*n* = 24), cats (*n* = 15), birds (*n* = 12), reptiles (*n* = 6), guinea pigs (*n* = 5), rodents (*n* = 1), fish (*n* = 5), miniature horses (*n* = 5), and petting zoo/farm animals (*n* = 7). While most facilities had multiple species visiting, three only allowed dogs, and a further three allowed only dogs or cats. Metropolitan and regional facilities had the lowest rate of allowing visiting animals, but no other demographic related to the decision to allow animals to visit a facility (*p* > 0.05). The range of animals allowed to visit RACF was also noted in the manager interview, exemplified by the following quote “happy to have a horse come in, or donkey come in or, you know, people with snakes or whatever we’ll, we’ll [sic] have anything come in for a short period of time”.

The frequency of visits varied, with 25% having daily visits and 52% having weekly visits; the remaining were monthly or less. Nearly half of respondents (*n* = 13, 42%) indicated that the most common reason for an animal to visit their RACF was ‘accompanying a friend or family member to visit a specific resident’. Other respondents indicated the animal visited due to being part of a physical or social therapy program for residents (*n* = 9, 29%) or accompanying a staff member to work (*n* = 2, 7%). Two authors examined free-text responses to a question asking how visiting animals were restricted within their RACF, and common approaches were identified. The most common approach was some form of ‘control’ of the animal by the handler (*n* = 14, 45%), primarily using a leash (*n* = 11). However, it is unclear how this would apply to some of the species allowed into RACF, e.g., reptiles. Animal hygiene (e.g., cleaning up after *n* = 3) and husbandry (e.g., vaccinated *n* = 6) needs were noted by some facilities; however, many (*n* = 5) had no additional restrictions for animals or handlers. In the interview with the manager similar information was provided where they explained that “the dog should be on a lead unless it’s such a well behaved or that because I do have some people who have got very highly trained dogs where they wouldn’t step an inch out of kilter. So, most dogs are on leashes.” and “[staff members] bring their cats that are either on leashes or dare I say it, they’re in baby pushers.”

Participants were asked to indicate (Yes/No/Unsure) whether an animal would be admitted to their facility across a range of potential scenarios related to animal health and hygiene (see Table 3 Many respondents did not answer this question despite continuing with the survey, the results need to be treated with caution. Interestingly, only five facilities required an animal to be bathed within 24 h of a visit, given that bathing reduces allergen and zoonotic transfer [36].

While the interviewed manager explained: “so long as your dog is healthy, you know, two or three days before you bring them in, it’s no sense in bringing in a dog that, you know, is maybe showing signs of being unwell with diarrhea or vomiting or stress or anything like that”. The manager also advised that there was no specific policy to restrict animals’ access to the facility in these situations.

### 3.2. Live-In Animals

Of the 31 respondents, just over half of the managers indicated that ‘live-in’ animals were allowed in their facility *(n* = 18, 58%); six facilities did not allow live-in animals (19%), and seven respondents did not respond to this item (23%). There were no significant demographic differences between facilities that did or did not allow live-in animals. When asked what types of animals were permitted and who owned them (either facility or resident), cats, birds, and fish/other aquatic species occurred evenly between the facility and resident ownership. However, dogs were predominantly resident-owned, while farm animals were exclusively facility-owned. Eighty-three per cent of these facilities (*n* = 15) noted that they prevented animal contact with staff or residents who have allergies or do not want contact. Only one respondent noted an instance of animal injury/illness in the past 12 months, and respondents reported no other cases of animal harm or zoonoses. However, the manager’s experience working in a psychogeriatric facility was quite different: “I have actually seen psychiatric aged care residents break into a birdcage and start eating the budgies… I’ve certainly seen more than enough residents swallowing goldfish faster than then you could feed them their pill” and when discussing their current facility, “Unfortunately, occasionally a chicken gets too stressed and passes away”. When queried about any restrictions for animals staying at their RACF, respondents most commonly identified utilizing some form of ‘temperament testing’ (*n* = 13, 72%). Animals were also restricted based on species (*n* = 9), weight (*n* = 1), reproductive (*n* = 6) or vaccination status (*n* = 9). Importantly, no respondents indicated that animals were restricted based on their age.

Eleven (61%) facilities with live-in animals allowed individual/resident ownership of animals. Where individual ownership was allowed, husbandry was primarily the resident’s responsibility (*n* = 8, 73%); for the remainder, the responsibility co-resided with staff and residents. Overwhelmingly individually owned animals were allowed contact with other residents (*n* = 10, 91%) and 73% (*n* = 8) facilities had a written plan in place should the animal outlive the owner. However, the manager we interviewed did not allow live-in animals, citing problems with re-homing the animal if the owner predeceased the pet (Supplementary Materials). Although not statistically significant, there was an apparent trend whereby private for-profit facilities were more likely to allow individually owned animals; no other demographic variables appeared to relate. Animals owned by the RACF were sourced via shelters (*n* = 4), breeders (*n* = 5), residents (*n* = 6), staff (*n* = 5), or unknown/stray (*n* = 9). Most commonly, facility-owned animals saw a vet annually (*n* = 9). Four facilities indicated resident birds (*n* = 3) or fish (*n* = 1) that did not access veterinary care.

When asked if facilities required additional information about having animals as part of their facility, managers demonstrated an interest in obtaining additional information on all aspects of having animals as part of their facility. When asked about general animal care guidance, 45% of managers wanted additional information; responses were similar for species-specific guidance (42%), recommended vet care (52%), specific advice about when animals “should not” have contact with humans (48%), and recommendations for particular screening (42%). Several also added free-text responses requesting additional training around managing animals left behind after an owner passes and policy/guideline development information. Only 10% of participants had no additional educational needs.

The Supplementary Materials present themes Newton et al. (2021) identified and demonstrates how the survey (shaded row) and interview data (quotes in italics) fit within those themes. The overarching issue of “animal-related policy” gaps/need for policy proved relevant to resident-/facility-owned and visiting animals, with the manager highlighting the implications of providing care for facility-owned animals and placing responsibility for the control and hygiene implications of visiting animals solely on the handler/owner of the animal. As noted earlier, “hand hygiene” policies were less prevalent than expected. The manager interview also suggested that this was not universally enforced (e.g., ad hoc treats available on the desk, presumably for hand feeding) and potentially only after handling animals. This could be problematic for animal encounters happening after a meal. The presence of food residue (or odour) may promote licking/excitement, elevating the risk of injuries and/or zoonotic transfer. “Behavioural assessment” of animals appears to range from certified/trained and formally assessed therapy dogs to ad hoc ‘well-behaved’ pets. The latter approach is the most frequent across the survey and the interview. “Health screening” and vaccination checks (visiting animals) were mentioned by approximately half of the surveyed facilities.

In contrast, a more general ‘wellness’ focus was mentioned in the interview. “Allergy/phobia screening and restricting access to certain areas” themes were not well-evidenced in the survey or interview, beyond the general acknowledgement of the need to keep animals away from those not keen on the interaction. While some facilities “restricted the species” allowed to visit, this does not seem to be the standard approach, with the interviewee suggesting that a wide range of domesticated species are supported. “Animal welfare” was one theme where the survey responses and interview ‘lived experience’ diverged markedly. While survey responses did suggest that animal welfare considerations were limited, this was accompanied by little to no adverse events in the past 12 months. In contrast, the interview provided multiple instances where animals were harmed and/or displayed concerning behavioural patterns due to activities within RACF.

## 4. Discussion

This mixed-method study aimed to examine RACF managers’ understanding of the risks associated with including animals in the RACF environment and the presence of RACF-specific animal policy around seven key themes. Overall, the results supported the hypothesis that Australian RACFs would have some form of policy guiding the incorporation of animals within their facilities. While animal policy coverage was found to be higher than that reported in previous international studies (e.g., [27,28,29], the content of the Australian policies (similar to the international studies) appears inadequate to protect either RACF residents or the animals visiting/residing with them. This lack of policy content is of concern from both a One Health and a One Welfare perspective.

### 4.1. One Health

Combining the One Health Framework and the Settings Approach, where RACF are a specific setting for the provision of AAA, this study’s results and findings demonstrate a risk to the animals and the residents. While there is national mandatory reporting of specific incidents, such as pressure injuries and falls [37], not all incidents in RACF are tracked nationally and identifying animal-related incidents from state department records is difficult. For example, Queensland Health only identified RACF in their admission coding in 2020. Previously, someone admitted from an RACF would be coded as being admitted from home, and therefore invisible when collecting RACF incident data (personal communication Qld Health [38]). Without accurate data, it is impossible to assess if the increasing popularity and inclusion of AAA in all its various forms has led to increased animal-related incidents in RACF. As Nhongo et al. [39] demonstrate, the frequency of incidence is affected by staffing levels and the time of day, where weekends, public holidays, and out-of-hours had higher rates of adverse events (falls, medication errors, skin tears etc.). However, it is unclear if the presence of animals during these times has or would result in additional adverse incidents.

Handwashing (or hand hygiene) is a cornerstone of infection control in any setting, and during the COVID-19 pandemic it was extensively promoted [40,41]. However, not all facilities surveyed had a handwashing policy specific to including animals in RACF; this lack is concerning. This lack of policy becomes increasingly problematic when raw-fed animals are permitted to visit because handwashing reduces the risk of foodborne illness [42,43]. As noted earlier, feeding raw meat diets to dogs and cats is associated with foodborne illnesses such as *Salmonella* spp., *Escherichia coli* (*E. coli*), and *Campylobacter* [44,45]. Even human-grade meat sources can carry these zoonotic bacteria [46,47]. IAHAIO recently released a position statement stating that animals fed a raw meat diet (or residing in a household with another animal fed raw food) should not participate in any AAI for 90 days from their last exposure, suggesting that exposure to raw diets poses considerable risk to humans interacting with these dogs [48]. Therefore, there is a need to develop and enforce handwashing practices, ideally before (to reduce the temptation for animals to lick residents’ hands) and after handling dogs and cats, especially when they may eat a raw diet or receive raw/dried meat treats. Similarly, the presence of reptiles, rodents, poultry, and other species that commonly carry *Salmonella* spp. is increasingly problematic when coupled with a lack of handwashing policy [43,49].

There are also zoonotic risks associated with interaction with very young animals (which were permitted to visit by all managers); the interviewed manager brought his puppy to the facility and made mention of young farm animals like chickens. Also, species other than dogs and cats were allowed by most managers; e.g., the interviewed manager admitted any animal “for a short time”. Besides young animals being more likely to scratch or bite residents because of their unpredictable behaviour, they are also more likely to carry zoonotic bacteria such as *Campylobacter* and *Giardia,* which can cause severe diarrhoea and vomiting [49,50]. Therefore, RACF should consider the risks and develop policies, e.g., an age restriction for visiting animals, which is not only a health issue but also a welfare issue. Interestingly, when Serpell et al. [51] surveyed AAI organizations, they reported that most only allow dogs over the age of 12 months to participate in visits. The contrast between Serpell et al.’s findings and what appears to be happening in Australian RACF may be due to a large number of ‘unregulated’ (i.e., not AAI-certified animal-handler teams) visits reported.

### 4.2. One Welfare

Similar to One Health, the One Welfare framework recognizes the ties between human and non-human welfare [24]. Providing AAA within RACF respectfully and through fostering animal welfare, human welfare is also supported; e.g., stressed animals are more likely to shed zoonotic disease and react aggressively to handling [24]. Instrumental use of animals (such as the ‘use’ of visiting fish in small plastic ponds so residents can go fishing [52]) may also result in decreased empathy, an unsuitable characteristic for anyone interacting with residents of an RACF [53]. Our findings, like those from international studies, suggest that animal welfare implications are not currently being considered sufficiently by RACF when arranging/facilitating AAA. This is not to suggest that animal welfare is being deliberately ignored, but more that RACF managers do not know what they do not know. Many of the respondents indicated a need for more information and guidance on the inclusion of animals with RACF. Within the broader field of human–animal studies, the welfare of animals within human–animal interactions is a relatively recent research focus with very limited exploration specific to the setting of RACF [9]. Further research into visiting-animal welfare is needed, ideally with acknowledgement of the wide array of animals beyond dogs taking part.

Most managers allowed “unregulated companion animals” to be brought in by community lay members, presumably to visit a family member or friend resident in the RACF. Many of these animals would have little training or socialization to enter an RACF, a strange environment full of unusual smells and equipment. While these unregulated animals were also identified by Stull, Hoffmann, and Landers [27], they have been omitted from much of the AAI research literature to date with an almost sole focus on ‘certified’ animal-handler teams [54]. Animals (mainly dogs) and their handlers accredited to visit RACF or other therapeutic venues undergo training and regular certification (e.g., [55]). These trained teams have an orientation to the environment, and the handlers receive education in animal behaviour. They are aware of the signs of stress in their animal and can intervene appropriately [56,57]. A layperson may not identify these stress signals, and staff may not be aware of signs of stress; a situation likely exacerbated by the variety of species visiting RACF [58]. Therefore, facilities allowing the entry of unregulated animals (aka noncertified animal-handler teams) should consider how to safely facilitate these visits for the wellbeing of residents and the animals concerned.

Also associated with unregulated animals is the inclusion of very young animals such as puppies and ‘baby’ farm animals. Calves, lambs, piglets and kids are often part of travelling farms brought to RACF. While these may be ethically sourced animals, there is the potential to create a new industry to produce juvenile farm animals for display. How these humanized farm animals are re-homed once their short-lived career as display animals is over is currently unclear. Returning them to a herd situation is unlikely, and there would be a limited number that could be possibly re-homed as pets. Including puppy and kitten visits would require careful handling and management from a human and animal perspective; as mentioned earlier, young dogs and kittens are more likely to scratch and bite. Puppies have critical socialization periods, when a frightening incident may leave them unsuitable for engaging in an RACF environment long term [59].

One Welfare concerns are also possible with animals that reside permanently within the RACF environment, either as facility-owned resident animals or personal pets. Not all facilities allow live-in animals; some animals may not be suitable to live in an RACF, due to size or lack of early socialization. In Australia, pet owners with suitable pets may have the option to keep their animal when moving into an RACF, however, they may need to pay an additional service fee allowed under the Extra Services Principles [57]. Those who cannot pay the extra fees and/or have a pet deemed unsuitable, due to size or facility rules, are faced with the difficult decision of what to do with their pets when they enter care. As a result, older pet owners may avoid seeking healthcare and delaying or refusing entry to RACF for fear of leaving their pet, who might face re-homing or euthanasia [60,61]. For those who can take their pets with them to RACF, managers identified the problem of pets whose owner predeceases them, with these issues presented as one of the reasons resident-owned animals were not permitted. The problem of owners predeceasing their pets was identified 30 years ago by Smith et al. [62] with seemingly little resolution since. Even when pets are allowed, and the facility is willing and able to re-home the animal should the owner predecease their pet, such a change may not suit the animals concerned. As a result, the new owner or the animal may become distressed, leaving the animal with an uncertain future [30].

While euthanasia appears to be the most unacceptable outcome for animals in RACFs, this is not the case. The manager interview highlighted several preventable incidents where animals died during interactions with residents. One incident involved overhandling of newly hatched chickens resulting in several chicks’ death and the incubators’ removal. The death of an animal during the provision of an AAA is not an acceptable outcome, regardless of whether the animal is a dog or a canary, a kitten or a fish [63]. A set of guidelines for appropriate animal interactions may have saved the animals, and prevented animal, staff, and resident distress. The guideline’s content could include information concerning the zoonotic risk of young poultry and their sensitivity to handling. Facilities could improve animal welfare by having older, friendly chicken breeds for supervised handling and only allowing observation of hatchlings.

### 4.3. Need for an Animal Policy as a Requirement

As mentioned earlier, most facilities had policies concerning animals. However, the content was lacking, may be linked to a lack of Federal Government guidance and no requirement for animal-specific policies in RACF [32]. Only Queensland Health has a readily available set of guidelines discussing animals visiting or residing in RACF. While the guidelines do discuss animals in the aged care setting, this mention is brief and almost exclusively directed at healthcare (hospital) facilities, whose focus (acute care) is not the model of care discussed here [64,65]. With this caveat in mind, it is interesting to note that the guidelines specify that only adult “domestic companion animals suitable as household pets” are allowed in RACF, and does not reflect the actual occurrence of animal visitations to, or residence in, RACF in Australia [64]. So, while RACF continue to provide AAA in various forms, there is little government guidance on how to best address the potential risks of such interactions.

## 5. Limitations

The primary limitation of this study is the sample size for the survey and the interview. However, as noted previously, the spread of RACFs across various jurisdictions matches the background population of Australian RACFs, and while our sample is small, it matches other Australian representative RACF studies (for example, [35,66]). Although these findings need to be treated with some caution, we are confident that the findings’ overall pattern is robust/generalizable. It is also important to acknowledge two broader issues that may have impacted recruitment and response numbers. Firstly, intense scrutiny of the RACF sector has been ongoing since the Royal Commission on the Quality and Safety in Aged Care (a three year examination of the entire Australian residential aged care sector); results were handed down in March 2021 [67]. As a result, the federal legislation surrounding the provision of care in RACF is likely to change considerably, resulting in additional work for managers and increased scrutiny of the practices of RACFs. The COVID pandemic further exacerbated this situation; ongoing lockdowns of many RACFs saw residents increasingly isolated and public scrutiny of infection control heightened further [68]. Given this, it is not surprising that the response rate was low. Future research on this topic would need to consider how to overcome participation reluctance, possibly by securing RACF industry endorsement and conducting in-person interviews. However, the size and distance between towns and cities in Australia makes conducting in-person interviews prohibitively expensive and time consuming. Conducting only one interview was not ideal, and future studies will need to seek broader engagement by managers of RACF; however, the interview provided valuable information concerning animal welfare that was not gathered in the survey, so we chose to include it here.

## 6. Conclusions

Similar with international findings, the current study suggests that facilities lack the necessary base policies to manage the inclusion of animals in RACF safely and in a manner that minimizes the potential for adverse outcomes for both human and non-human participants in this unique health setting. Our study has shown that current reliance on AAI providers to keep animals and residents safe is not always applicable, with most visiting animals arriving through sources other than an AAI organization, potentially jeopardising both human and animal welfare. While the reported number of adverse incidents is low in this study and internationally [27,29], this may be due to reporting pathways or previously low(er) rates of animal inclusion. With the increasing presence of animals in RACF, further research and development of national guidelines linked to aged care policy, with training and education packages for RACF staff, is needed to ensure the wellbeing of all concerned.

## Figures and Tables

**Table 1:** Demographic Data.

Funding Source	*N*	%
Private for profit	14	45%
Non-profit	17	55%
Remoteness		
Modified Monash	*N*	%
1 Metropolitan	12	39%
2 Regional center	7	23%
3 Large rural town	4	13%
4 Medium rural town	3	10%
5 Small rural town	4	13%
6 Remote	1	3%
7 Very remote	0	0%
Size		
Number of beds	*N*	%
0–30	2	7%
31–60	11	36%
61–100	7	23%
101–180	7	23%
>180	4	13%

**Table 2:** Policy provision by animal source.

	Facility-Owned Animals *	Resident-Owned Animals *	Visiting Animals
	Number (%)	Number (%)	Number (%)
Our policy includes instructions for hand hygiene practices for staff and residents who interact with this type of animal.	9 (32%)	8 (29%)	15 (48%)
Our policy includes information on training or other educational activities for staff members involved in this type of animal care.	4 (14%)	4 (14%)	6 (19%)
Our policy includes a description of specific areas in the Residential Aged-Care Facility where this type of animal is/is not permitted.	8 (29%)	7 (25%)	12 (39%)
Our policy includes a procedure to follow if this type of animal bites a person (staff member or resident).	6 (21%)	5 (18%)	8 (26%)
Our policy includes a procedure to follow if this type of animal becomes ill or injured.	4 (14%)	4 (14%)	5 (16%)
Our policy includes specific regulations about what species this animal can be.	4 (14%)	1 (4%)	7 (23%)
Our policy specifically designates who is responsible for this type of animal’s care, including maintaining veterinary records	9 (32%)	8 (29%)	12 (39%)

* Percentage calculated on the 28 facilities allowing resident- or facility-owned animals.

**Table 3:** Animal health and hygiene scenario-based questions “Would you permit an animal to visit if they had…?”.

Animal Has/Had	Yes	No	Unsure
Current diarrhea	0	13	0
Diarrhea in the past 2 weeks	0	10	3
Not bathed in the past 24 h	8	5	0
Vomited in the past 2 weeks	1	9	3
Vomited in past 24 h	0	13	0
Appears sick	0	13	0
No vet check in the past 7 days	11	2	0
Current antibiotic prescription	3	8	2

## Data Availability

Supporting data is hosted on Central Queensland University’s secure server and is available by application to the corresponding author. The data is not publicly available due to ethical considerations.

## Supplementary Materials

The following supporting information can be downloaded at: https://www.mdpi.com/article/10.3390/ani12243591/s1, A composite of Quantitative and Qualitative results using themes identified in Newton, et al. [9].
