# Supplementary material for: Fur, Fin, and Feather: Management of Animal Interactions in Australian Residential Aged Care Facilities
Source: Animals (Basel). 2022 Dec 19;12(24):3591. doi: 10.3390/ani12243591 (PMC9774757; doi:10.3390/ani12243591)
Supplement: Supplementary file 1 [file animals-12-03591-s001.zip › animals-2078430-supplementary.pdf]

animals

MDPI

A composite of Quantitative and Qualitative results using themes identified in Newton, et al. [1].

	Facility Owned	Resident Owned	Visiting
Animal policies as a requirement	87%	81%	84%
Manager paper mentions=3	*We had an industrial issue with the staff and the staff refused to actually look after the dog, so it was the housekeeper's job to ensure that the dog was fed, washed, taken for walks, etc, etc. Because I couldn't trust the staff to look after the dog, I couldn't trust the staff to look after the fish tanks. So, we had to outsource someone to come in and clean the fish tank, maintain the fish tanks and so on*	*[not allowed] .. and the main reason being is who's going to look at the fish, and you can guarantee the resident passes away the family will want us to take the fish and we just can't do that*	*Why did the dog bite someone if the dog wasn't under control? Let's not blame the dog. Let's blame the person who's on the end of the leash if the dog makes a mess on the carpet or on the floor*
Hand hygiene	32%	29%	48%
mentions=2			*sitting on my desk is two types of dog treats*<br>*[it] comes down to whoever handles a pet, should go off and wash their hands and so on. You know, we usually do it at morning, after morning tea or after lunch. So, people have opportunities to leave the setting and go and wash their hands and that sort of thing*
Animal behavioural assessment	n/a	n/a	72%
mentions=8			*we have is we had numerous pets come visit*

	Facility Owned	Resident Owned	Visiting
			we had any amount of therapy dogs who have got the certificate and come in with their little coats<br><br>*have staff who have particular dogs that are quite calm around different people and so on.*<br><br>*I brought my four-month-old miniature poodle into show off*<br><br>*encourage staff to bring their pets in for the day*<br><br>*We do have McDonald's farm come*<br><br>*about the size of the dog the type of dog. And to so long as it's friendly. Bringing in a dog that you can approach*<br><br>*the dog should be on a lead unless, it's a such a well behaved or that. Because I do have some people who have got very highly trained dogs where they wouldn't step an inch out of kilter. So, most dogs are on leashes*
Animal Health screening and feeding for raw diets	Vets visit at least yearly 14<br>No vet 4 (birds x 3, 1xfish)	82% (preventative medication)	54% (Vaccinations checked)
mentions=3	*what is the dog actually eating*		*so long as your dog is healthy, you know, two or three days before you bring them in. It's no sense in bringing in a dog that, you know, is maybe showing signs of being unwell with diarrhoea or vomiting or stress or anything like that*

	Facility Owned	Resident Owned	Visiting
			it comes down to the control and what you put down as ensuring that dogs clean don't bring in a mangy dog or a dog infected by fleas, things like that
Allergy and phobia screening	83% (across resident and facility owned we did not ask how)		Not asked
mentions=1			*certainly not everybody wants to see the pets, you know, there are those that don't like dogs, or those who don't believe that dogs should be inside*
Restricted Zones	29%	25%	39%
mentions=2	*that dog used to come and hide under my desk in my office just to get away from people*		*And under control*
Species restriction	14%	4%	23% had a policy restricting species, however dogs, cats, birds, reptiles, guinea pigs, rodents, fish, miniature horses, and petting zoo/farm animals were all permitted
mentions=5	*Food Standards came in, you weren't allowed to use eggs that were not processed through a proper egg processing plant*<br>*But again, Chook's and Chook food invite rats, mice, snakes*<br>*I don't believe cats are suitable in an aged care facility. Because of the need to sort of go outside and the outside and that sort of thing*		*We do have McDonald's farm come*<br>*[We are] happy to have a horse come in, or donkey come in or, you know, people with snakes or whatever we'll, we'll have anything come in for a short period of time*
Animal welfare	14% provided education for staff on caring for FO animals.	14% provided education for staff on caring for RO animals.	19% provided education for staff on caring for Visiting animals.

	Facility Owned	Resident Owned	Visiting
	32% had a policy designating the person responsible for the care of this type of animal.	29% had a policy designating the person responsible for the care of this type of animal.<br>From the free text responses: "Obedience training for dogs whose owners have deceased while in care and when other family members are unable to re-home them. To keep them at the facility to be managed by staff or other residents can often be an issue if the person who has trained them is no longer around.	39% had a policy designating the person responsible for the care of this type of animal.<br>42% allowed animals accompanying a friend or family member.
mentions=12	*would sort of annoy the animals, or the fish or birds to such a degree that they either died because of the stress or whatever*<br>*they would eat them. And I have actually seen psychiatric aged care residents break into a birdcage and start eating the budgies*<br>*I've certainly seen more than enough residents swallowing goldfish faster than then you could feed them their pills*<br>*that dog used to come and hide under my desk in my office just to get away from people*<br>*what is the dog actually eating*<br>*is the dog actually getting exercise*<br>*stress that dogs go through. When residents start passing away. Now, you can actually see it in the dog. And believe it or not, I actually believe between the dogs and*		*Unfortunately, occasionally a chicken gets too stressed and passed away*<br>*We have staff members who bring in their cats, their cats are either on leashes, or dare I say it they're in. Baby pushers, and so on*<br>*[It] comes down to whoever handles a pet, should go off and wash their hands and so on. You know, we usually do it at morning, after morning tea or after lunch. So, people have opportunities to leave the setting and go and wash their hands and that sort of thing.*

	Facility Owned	Resident Owned	Visiting
	the cats in an aged care facility. They will spend time with people who are dying<br><br>*an onsite animal wouldn't be able to handle that turnover*<br><br>*actually, outsourced all the care of the dog to a contractor*		

References

1. Newton, W.; Signal, T.; Judd, J. The guidelines and policies that influence the conduct of Animal-Assisted Activities in Residential Aged-Care Facilities: A systematic integrative review. *Complement Ther Clin Pract* **2021**, *44*, 101395, doi:10.1016/j.ctcp.2021.101395.
